# A nomogram model incorporating blood biomarkers predicts 3-week functional outcomes in stroke patients

**DOI:** 10.3389/fnins.2025.1609156

**Published:** 2025-05-20

**Authors:** Suzhen Ye, Ting Ding, Xin Gao, Xuezhen Zhou, Meihong Xiu, Yu Xia

**Affiliations:** ^1^The First Affiliated Hospital of Wenzhou Medical University, Wenzhou, China; ^2^Qingdao Mental Health Center, Qingdao, China; ^3^Beijing Huilongguan Hospital, Peking University Huilongguan Clinical Medical School, Beijing, China

**Keywords:** stroke, activities of daily living, nomogram, biomarkers, prediction

## Abstract

**Objective:**

Accurate prediction of functional outcomes of stroke remains clinically challenging. The present study was designed to identify baseline biomarkers in demographic, clinical data, and blood biomarkers to predict 3-week outcomes in stroke patients.

**Methods:**

A prospective cohort of two hundred patients with stroke was recruited at the hospital and followed for 3 weeks. We applied the Barthel Index (BI) to measure the activities of daily living functions in stroke patients. The good outcome or poor outcome groups were classified based on the BI scores. A logistic regression analysis was performed to identify independent predictors, which were subsequently integrated into a nomogram. Discrimination and calibration values of the nomogram were analyzed, and its utility was assessed using decision curve analysis.

**Results:**

Four blood biomarkers, including PT (OR = 1.45, 95% CI: 1.05–2.12), FIB (OR = 1.49, 95% CI: 1.14–2.00), RBG (OR = 1.20, 95% CI: 1.03–1.40), and UA (OR = 1.00, 95% CI: 0.99–1.00) were independent predictors of the 3-week functional outcomes after a stroke. The nomogram incorporating these biomarkers demonstrated moderate discriminative ability (AUC values = 0.714, 95%CI: 0.641–0.786), with satisfactory calibration and positive net benefit on DCA across clinically relevant threshold probabilities.

**Conclusion:**

We developed a pragmatic nomogram integrating readily available blood biomarkers to predict 3-week functional outcomes in stroke patients. While validation in larger cohorts is warranted, our findings provide new evidence in early risk stratification and personalized rehabilitation planning, potentially improving post-stroke care efficiency.

## 1 Introduction

Stroke is a serious health issue with significant global impacts. It affects 15 million patients worldwide each year. The effects of a stroke vary from mild to severe and may lead to long-term disabilities in 2/3 of patients or even death in approximately 1/3 of patients (Adamson et al., 2004; Johnston et al., 2009). About 80% of strokes are ischemic and occur under the age of 50 years (Maaijwee et al., 2014). Prolonged hospitalization for stroke treatment results in a significant increase in healthcare costs. Rehabilitation for stroke sequelae is also a long-term and potentially costly process, including physical therapy, occupational therapy, speech therapy, etc. However, despite significant advances in basic research, there is a lack of reliable blood biomarkers to predict prognosis in this patient population (Whiteley et al., 2009).

Patients who have experienced a stroke typically face a range of motor, sensory, language, cognitive, and psychological disorders that can significantly impact their self-care abilities and overall quality of life (Anderson et al., 1995; Kalra and Langhorne, 2007; Winstein et al., 2016). The aftermath of a stroke can lead to long-term disability and affect a person’s ability to function independently (Kalra and Langhorne, 2007). Post-stroke evaluation is critical for optimal stroke care. Currently, not all patients with stroke achieve the same level of recovery, even after hospitalization and intensive rehabilitation (Jørgensen et al., 1997). Stroke survivors face many challenges that may affect their ability to care for themselves and maintain a good quality of life. A study in stroke survivors found that 5 years after stroke, a large proportion of participants reported impaired of health-related quality of life (HRQoL), mainly related to pain and discomfort (Segerdahl et al., 2023). The study found that higher age and longer hospitalization were predictors of impaired HRQoL related to mobility, self-care, and daily activities. These findings highlight the importance of identifying patients at a high risk for poor HRQoL, who may benefit from specialized attention and psychological support.

The early prediction of prognosis in patients with stroke, including the long-term functional outcomes in patients, is of great interest. In clinical practice, once a diagnosis of stroke has been confirmed, appropriate treatment must be administrated promptly to ensure maximum benefit to the patient and to prevent complications (Adams et al., 2003; Duncan et al., 2005; Winstein et al., 2016). Previous studies have observed a relationship between blood biomarkers and diagnosis and poor stroke outcomes (Whiteley et al., 2008; Welsh et al., 2009; Whiteley et al., 2009; Monbailliu et al., 2017; Lai et al., 2019; Amalia, 2021; Barba et al., 2023). For example, a study with 270 patients with stroke reported after controlling for stroke severity and age, IL-6 and N-terminal pro-brain natriuretic peptides correlated with poor outcomes at month 3 (Whiteley et al., 2012). Another study used demographic, clinical, and biochemical characteristics obtained within 2 days after strokes to develop models that accurately predict stroke mortality and morbidity at 90 days (Fernandez-Lozano et al., 2021). Although there have been many previous studies of candidate biomarkers, none have yet demonstrated sufficient sensitivity and specificity in studies with small sample sizes for routine management of stroke.

We hypothesized that an integrated approach that included biomarkers, demographic data, clinical presentations, and laboratory parameters measured in blood samples would improve the prediction of poor outcomes after stroke. A nomogram is a statistical tool that evaluates and calculates the precise risk of long-term outcomes for patients with stroke (Kim et al., 2016). Considering the complex underlying mechanism and varying prognosis biomarkers for functional outcomes after stroke, an effective nomogram model has not yet been established for patients with stroke (van Alebeek et al., 2018). Studies have shown that the Barthel index (BI) is a good functional outcome measure in patients with stroke and reported that BI scores of more than 40 at discharge can predict better outcomes (Mahoney and Barthel, 1965; Nakao et al., 2010). Also, a strong correlation between the BI scale and the National Institutes of Health Stroke Scale (NIHSS), another good scale to assess the severity of stroke and functional outcomes, has been reported (Bathla et al., 2023). Therefore, this study aimed to measure the demographic, clinical data, and laboratory indicators and then establish a predictive model to predict the 3-week outcome after strokes.

## 2 Methods

### 2.1 Subjects

This study was conducted from June 2021 to April 2022 in the First Affiliated Hospital of Wenzhou Medical University. A total of two hundred participants with stroke (male/female = 133/67) were retrospectively collected from the outpatient clinic. All subjects were admitted to the stroke units and received standard therapy, e.g., antiplatelet therapy and statin therapy. The dataset included demographic data, clinical data, medication details, laboratory measurements, and more.

The inclusion criteria were: (1) ≥ 18 years; (2) diagnosis of ischemic stroke; (3) clear consciousness and willingness to cooperate. The following are exclusion criteria: (1) a history of depression or other mental disorders based on medical records; (2) serious systematic diseases such as cancer; (3) with recent infections; and (4) unstable vital signs.

### 2.2 Assessments of activities of daily living (ADL)

The BI scale is a simple and quick test that helps to evaluate a patient’s level of independence in their daily activities. The BI scale comprised 10 items, including feeding, grooming, bathing, dressing, bowel and bladder care, toilet use, ambulation, transfers, and stair climbing. Each item was scored between 0 and 15, where 0 indicates the patient is completely dependent and 15 indicates complete independence. Higher scores indicate greater independence in ADL. All assessors completed a standardized training program prior to data collection. Inter-rater reliability was quantified using the intraclass correlation coefficient (ICC = 0.90) for total BI scores.

Patients were categorized into two subgroups, the good outcome group (>50 scores on the BI scale, GOG) or the poor outcome group (<50 scores, POG), based on the BI scores.

### 2.3 Blood measurements

Fasting venous blood samples were collected between 7:00 am and 8:00 am. After collection, blood samples are centrifuged to separate the serum. Differential blood counts were determined using an XN-3000 automated counter and white blood cell (WBC), neutrophil (NEU), and RBC red blood cell was measured. Hb Hemoglobin, Hct Hematocrit, MCV mean corpuscular volume, MCH mean corpuscular hemoglobin, MCHC mean corpuscular hemoglobin concentration, SD value of red blood cell volume distribution width, PLT platelet, PCT platelet accumulation, MPV mean platelet volume, PT prothrombin time, PTA prothrombin activity, FIB fibrin, APTT activated partial thromboplastin time, ALB serum albumin, GLO serum globulin, ALT/GPT glutamic pyruvic transaminase (GPT), AST/GOT Glutamic oxaloacetic transaminase, RBG random blood glucose, BUN blood urea nitrogen, Scr creatinine, UA uric acid, K calcium, NA natrium, CL chlorine, TC total cholesterol, TG triglyceride, HDL high-density lipoprotein, LDL low-density lipoprotein, APO-AI apolipoprotein AI, APO-B apolipoprotein B, Lpa lipoprotein B, CRP C-reactive protein, HCY homocysteine, CK creatine kinase, CK-MB creatine kinase isoenzyme, LDH lactic dehydrogenase were all measured in the laboratory of the hospital.

### 2.4 Statistical analysis

Statistical analyses were performed with the R software (version 4.1.3).

Continuous variables were described as mean ± SD and student t-tests between groups. The categorical variable was presented as n (%) and calculated by the *X*^2^ tests. Multivariate analysis was performed after further screening for variables with a *p* of <0.05 in the univariate analysis. Univariate and multivariable logistic regression analyses were used to analyze the related factors to stroke outcomes. The nomogram for predicting outcomes was established based on optimized multivariate logistic regression. The strength of correlations between related factors and functional outcomes was measured using the OR and 95% CI. The nomogram prediction models were validated based on bootstrapped calibration curves (1,000 times) and marginal R-squared (*R*2 M). This study used the ROC curve approach to test the optimized multivariate logistic regression model’s discrimination abilities. The decision curve analysis was conducted to examine the clinical benefit of the optimized multivariate logistic regression model.

## 3 Results

### 3.1 Patient characteristics in the GOG and POG groups

Patients were followed up after 3 weeks after stroke. At the end of the week 3 follow-up in this study, 82(41%) patients were considered as good outcomes, and 118 patients (59%) were as poor outcomes. No significant difference was observed in gender between the GOG and POG groups (*p* > 0.05).

The demographic characteristics, medical history, laboratory data, and position of the disease in all patients are shown in Table 1. We also compared the baseline demographic and clinical characteristics between the GOG and POG groups. The POG group had older age and a longer course of disease than the GOG group (*p* < 0.001). More patients in the GOG group had no history of stroke than those in the POG group (*p* = 0.009). In addition, left-sided hemiplegic stroke patients had a better outcome after stroke (*p* = 0.02).

**Table 1:** Comparison of baseline characteristics between the GOG and POG groups in the study population.

Variables	All samples	GOG groups (BI > 50) (*n* = 82)	POG groups (BI < 50) (*n* = 118)	*p*-value
Age	66.2 ± 11.9	62.1 ± 11.6	69.1 ± 11.4	<0.001
Sex, *n* (%)				0.18
Male	132 (66.0%)	59 (72%)	73 (61.9%)	
Female	68 (34%)	23 (28.1%)	45 (38.1%)	
Weight (Kg)	63.6 ± 10.3	63.9 ± 10.1	63.5 ± 10.4	0.79
Stature (m)	1.7 ± 0.1	1.7 ± 0.1	1.6 ± 0.1	0.35
BMI	23.4 ± 3.2	23.3 ± 3.2	23.4 ± 3.2	0.83
Marital status				0.32
Single	4 (2.0%)	2 (2.4%)	2 (1.7%)	
Married	187 (93.5%)	74 (90.2%)	113 (95.8%)	
Divorced	1 (0.5%)	1 (1.2%)	0 (0.0%)	
Widowed	8 (4.0%)	5 (6.1%)	3 (2.5%)	
Literacy levels				0.30
Itinerary	36 (18.0%)	11 (13.4%)	25 (21.2%)	
Primary	76 (38.0%)	31 (37.8%)	45 (38.1%)	
Junior	52 (26%)	22 (26.8%)	30 (25.4%)	
Senior	24 (12.0%)	10 (12.2%)	14 (11.9%)	
College	12 (6.0%)	8 (9.8%)	4 (3.4%)	
Smoking				0.34
No	119 (59.5%)	45 (54.9%)	74 (62.7%)	
Yes	81 (40.5%)	37 (45.1%)	44 (37.3%)	
Drinking				0.63
No	115 (57.5%)	45 (54.9%)	70 (59.3%)	
Yes	85 (42.5%)	37 (45.1%)	48 (40.7%)	
HP				0.19
No	40 (20.5%)	21 (25.6%)	20 (17.0%)	
Yes	159 (79.5%)	61 (74.4%)	98 (83.1%)	
DM				0.50
No	118 (59.0%)	50 (61.0%)	68 (57.6%)	
Yes	82 (41.0%)	32 (39.0%)	50 (42.3%)	
CAD				0.20
No	183 (59.0%)	78 (95.1%)	105 (89.0%)	
Yes	17 (8.5%)	4 (4.9%)	13 (11.0%)	
AF				0.37
No	185 (92.5%)	78 (95.1%)	107 (90.7%)	
Yes	15 (7.5%)	4 (4.9%)	11 (9.3%)	
Gout				1.00
No	188 (94.0%)	77 (94.0%)	111 (94.1%)	
Yes	12 (6.0%)	5 (6.1%)	7 (5.9%)	
HLP				1.00
No	183 (95.0%)	75 (91.5%)	108 (91.5%)	
Yes	17 (8.5%)	7 (8.5%)	10 (8.5%)	
History of stroke				0.009
No	162 (81.0%)	74 (92.0%)	88 (74.6%)	
Yes	38 (19.0%)	8 (9.8%)	30 (25.4%)	
Position of the disease				0.82
Cerebrum	145 (72.5%)	59 (72.0%)	86 (72.9%)	
Brainstem	43 (21.5%)	19 (23.2%)	24 (20.3%)	
Opisthencepalon	12 (6.0%)	4 (4.9%)	8 (6.8%)	
Hemiplegic side				0.02
Left	92 (46.0%)	46 (56.1%)	46 (39.0%)	
Right	104 (52.0%)	36 (43.9%)	68 (57.6%)	
Both	4 (2.0%)	0 (0.0%)	4 (3.4%)	
Course (d)	13.1 ± 9.2	11.2 ± 6.8	14.4 ± 10.3	0.008

BMI, body mass index; HP, hypertension; DM, diabetes mellitus; CAD, coronary artery disease; AF, atrial fibrillation; HLP, hyperlipidemia; d, days.

In terms of laboratory indicators, patients in the POG group had more WBC counts, PT, FIB, RGB, and BUN/Scr, as well as lower ALB, UA, and NA levels than those in the GOG group (all *p* < 0.05) (Table 2).

**Table 2:** Comparison of baseline characteristics between the GOG and POG groups.

Variables	All samples	GOG groups (BI > 50) (*n* = 82)	POG groups (BI < 50) (*n* = 118)	*p*-value
WBC (*10^9^/L)	6.66 ± 1.97	6.26 ± 1.69	6.93 ± 2.10	0.014
NEU (%)	0.67 ± 0.09	0.64 ± 0.09	0.68 ± 0.08	0.003
RBC (*10^12^/L)	4.21 ± 0.52	4.29 ± 0.57	4.15 ± 0.47	0.079
Hb (g/L)	128.57 ± 15.99	130.51 ± 17.30	127.22 ± 14.94	0.164
Hct (L/L)	0.38 ± 0.04	0.38 ± 0.05	0.38 ± 0.04	0.207
MCV (fl)	90.40 ± 4.60	89.84 ± 4.50	90.80 ± 4.64	0.144
MCH (pg)	30.62 ± 1.75	30.47 ± 1.66	30.72 ± 1.81	0.309
MCHC (g/L)	338.66 ± 8.41	339.23 ± 8.49	338.26 ± 8.38	0.426
RDW (%)	13.25 ± 1.05	13.31 ± 1.26	13.21 ± 0.87	0.508
SD value of RDW (fl)	43.73 ± 4.54	43.79 ± 3.85	43.70 ± 4.98	0.881
PLT (*10^9^/L)	245.29 ± 71.33	242.89 ± 69.49	246.97 ± 72.83	0.690
PCT (L/L)	0.24 ± 0.06	0.23 ± 0.06	0.24 ± 0.06	0.640
MPV (fl)	9.78 ± 1.03	9.77 ± 1.01	9.78 ± 1.05	0.912
SD value of PDW (fl)	16.03 0.36	16.03 ± 0.45	16.03 ± 0.29	0.918
Platelet-large cell ratio (%)	24.37 ± 7.15	24.33 ± 7.02	24.40 ± 7.27	0.946
PT(s)	13.62 ± 1.15	13.43 ± 0.74	13.75 ± 1.35	0.035
PTA (%)	100.11 ± 65.08	108.45 ± 100.11	94.31 ± 13.54	0.207
FIB (g/L)	3.92 ± 1.17	3.64 ± 1.08	4.12 ± 1.19	0.003
APTT(s)	36.56 ± 4.78	36.32 ± 4.38	36.73 ± 5.06	0.543
D-dime (mg/L)	0.81 ± 1.01	0.66 ± 1.00	0.92 ± 1.01	0.076
ALB (g/L)	38.19 ± 4.77	39.22 ± 4.32	37.48 ± 4.94	0.009
GLO (g/L)	29.93 ± 14.50	30.40 ± 22.17	29.60 ± 4.12	0.747
ALT (U/L)	38.55 ± 27.81	38.66 ± 20.28	38.47 ± 32.10	0.959
AST (U/L)	33.27 ± 18.29	32.18 ± 14.05	34.03 ± 20.76	0.455
RBG (mmol/L)	6.70 ± 2.21	6.20 ± 1.86	7.04 ± 2.37	0.006
BUN (mmol/L)	6.63 ± 5.16	6.38 ± 4.21	6.80 ± 5.74	0.557
Scr (umol/L)	72.27 ± 34.70	77.23 ± 43.77	68.82 ± 26.32	0.122
BUN/Scr (%)	22.51 ± 8.02	21.06 ± 6.68	23.52 ± 8.72	0.025
UA (umol/L)	287.46 ± 95.55	307.88 ± 100.32	273.26 ± 89.80	0.013
K (mmol/L)	3.93 ± 0.45	3.89 ± 0.47	3.95 ± 0.43	0.403
NA (mmol/L)	139.10 ± 2.85	139.59 ± 2.66	138.77 ± 2.94	0.043
CL (mmol/L)	103.30 ± 6.99	103.56 ± 2.57	103.12 ± 8.85	0.609
TC (mmol/L)	3.66 ± 0.88	3.64 ± 0.95	3.67 ± 0.83	0.779
TG (mmol/L)	1.65 ± 0.84	1.73 ± 0.97	1.60 ± 0.74	0.324
HDL-C (mmol/L)	0.92 ± 0.19	0.93 ± 0.19	0.92 ± 0.20	0.653
LDL-C (mmol/L)	2.24 ± 0.66	2.21 ± 0.68	2.26 ± 0.64	0.650
APO-AI (g/L)	1.46 ± 4.04	1.16 ± 0.16	1.68 ± 5.25	0.288
APO-B (g/L)	0.91 ± 1.30	0.85 ± 1.01	0.96 ± 1.48	0.551
Lpa (mmol/L)	279.3 ± 313.5	245.5 ± 263.3	302.8 ± 343.3	0.183
CRP (mg/L)	8.06 ± 13.7	5.74 ± 12.47	9.67 ± 14.36	0.041
HCY (umol/L)	12.13 ± 4.16	12.21 ± 5.12	12.08 ± 3.35	0.849
CK (u/L)	76.13 ± 69.92	78.44 ± 48.78	74.53 ± 81.61	0.673
CK-MB (u/L)	13.94 ± 7.80	13.98 ± 7.42	13.91 ± 8.08	0.951
LDH (u/L)	222.90 ± 66.9	214.9 ± 50.8	228.47 ± 75.88	0.130

WBC, white blood cell; NEU, neutrophil; RBC, red blood cell; Hb, Hemoglobin; Hct, Hematocrit; MCV, mean corpuscular volume; MCH, mean corpuscular hemoglobin; MCHC, mean corpuscular hemoglobin concentration; SD, value of red blood cell volume distribution width; PLT, platelet; PCT, platelet accumulation; MPV, mean platelet volume; PT, prothrombin time; PTA, prothrombin activity; FIB, fibrin; APTT, activated partial thromboplastin time; ALB, serum albumin; GLO, serum globulin; ALT/GPT, glutamic pyruvic transaminase (GPT); AST/GOT, Glutamic oxaloacetic transaminase; RBG, random blood glucose; BUN, blood urea nitrogen; Scr, creatinine; UA, uric acid; K, calcium; NA, natrium; CL, chlorine; TC, total cholesterol; TG, triglyceride; HDL, high-density lipoprotein; LDL, low-density lipoprotein; APO-AI, apolipoprotein AI; APO-B, apolipoprotein B; Lpa, lipoprotein B; CRP, C-reactive protein; HCY, homocysteine; CK, creatine kinase; CK-MB, creatine kinase isoenzyme; LDH, lactic dehydrogenase.

### 3.2 Factors associated with the 3-week outcomes after strokes

Univariate logistic analysis was constructed with the better or poor outcomes after stroke as the independent variable, and the demographic data and clinical characteristics as the dependent variables, including sex, age, weight, body mass index (BMI), marital status, literacy levels, smoking status, drinking, hypertension (HP), diabetes mellitus (DM), coronary artery disease (CAD), atrial fibrillation (AF), hyperlipidemia (HLP), Gout, history of stroke, position of stroke, hemiplegic site and course of disease. The results revealed that age (OR = 2.42, 95% CI:1.36–4.37), literacy levels (OR = 0.23, 95% CI:0.05–0.91), history of stroke (OR = 3.10, 95% CI:1.9–7.701), and course of the disease (OR = 1.06, 95% CI: 1.01–1.11) were associated with the outcomes after stroke in patients (all *p* < 0.05) (Table 3).

**Table 3:** Univariate logistic regression analysis for 3-week outcomes with demographic and clinical data as independent variables.

Variable		*n*	*OR* (95% *CI*)	*p*-value
Sex	Male	132	Reference	
	Female	68	1.57 [0.86;2.93]	0.142
Age	<65		Reference	
	≥65		2.42 [1.36;4.37]	0.003
Weight (kg)		200	1.00 [0.97;1.02]	0.787
Stature (m)		200	0.16 [0.00;8.12]	0.362
BMI (Kg/m^2^)		200	1.01 [0.92;1.10]	0.827
Marital status	Single	4		
	Married	187		
	Divorced	1		
	Widowed	8		
Literacy levels	Ittiteracy	36	Reference	
	Primary	76	0.64 [0.27;1.49]	0.306
	Junior	52	0.61 [0.24;1.48]	0.274
	Senior	24	0.62 [0.21;1.86]	0.394
	College	12	0.23 [0.05;0.91]	0.037
Smoking	No	119	Reference	
	Yes	81	0.72 [0.41;1.29]	0.272
Drinking	No	115	Reference	
	Yes	85	0.83 [0.47;1.48]	0.536
HP	No	41	Reference	
	Yes	159	1.68 [0.84;3.39]	0.143
DM	No	117	Reference	
	Yes	83	1.09 [0.61;1.95]	0.767
CAD	No	183	Reference	
	Yes	17	2.35 [0.78;8.85]	0.132
AF	No	185	Reference	
	Yes	15	1.95 [0.63;7.50]	0.256
Gout	No	188	Reference	
	Yes	12	0.96 [0.29;3.46]	0.952
HLP	No	183	Reference	
	Yes	17	0.99 [0.36;2.87]	0.979
History of stroke	No	162	Reference	
	Yes	38	3.10 [1.39;7.70]	0.005
Position of the disease	Cerebrum	145	Reference	
	Brainstem	43	0.87 [0.43;1.74]	0.684
	Opisthencephalon	12	1.35 [0.40;5.43]	0.643
Hemiplegic side	Left	92		
	Right	104		
	Both	4		
Course (d)		200	1.06 [1.01;1.11]	0.016

BMI, body mass index; HP, hypertension; DM, diabetes mellitus; CAD, coronary artery disease; AF, atrial fibrillation; HLP, hyperlipidemia; d, days.

Then the univariate logistic analysis was also performed with the laboratory indicators as the dependent variables, including WBC, FIB, ALB, RBG, BUN/Scr ratio, UA, Hb, Hct, MCV, MCH, MCHC, RDW, PLT, PCT, MPV, PT, PDW, PTA, APTT, D-dime, GLO, ALT, AST, RBG, BUN, Scr, BUN/Scr, K, NA, CL, TC, TG, HDL-C, LDL-C, APO-AI, APO-B, Lpa, CRP, HCY, CK, CK-MB, and LDH.

Multivariate logistic regression analysis showed that a few factors were not influential on stroke outcomes, to further accurately explore the risk factors, this study removed no significant factors (*p* > 0.05) until the *p*-value of all factors was less than or equal to 0.05. The results demonstrated that PT (OR = 1.45, 95% CI:1.05–2.12), RBG (OR = 1.20, 95% CI:1.03–1.40), BUN/Scr (%) (OR = 1.04, 95% CI:1.00–1.08), and UA (OR = 1.00, 95% CI:0.99–1.00) were correlated with the stroke outcomes in patients (all *p* < 0.05) (Table 4).

**Table 4:** Univariate logistic regression analysis for 3-week outcomes with blood biomarkers as independent variables.

Variable	*n*	*OR* (95% *CI*)	*p*-value
WBC (*10^9^/L)	200	1.21 [1.03;1.42]	0.020
N (%)	200	1.68 [5.86;4,811]	0.003
RBC (*10^12^/L)	200	0.60 [0.34;1.04]	0.071
Hb (g/L)	200	0.99 [0.97;1.00]	0.153
Hct (L/L)	200	0.01 [0.00;8.96]	0.194
MCV (fl)	200	1.05 [0.98;1.12]	0.151
MCH (pg)	200	1.09 [0.92;1.28]	0.317
MCHC (g/L)	200	0.99 [0.95;1.02]	0.423
RDW (%)	200	0.91 [0.69;1.19]	0.481
SD value of RDW (fl)	200	1.00 [0.93;1.06]	0.886
PLT (*10^9^/L)	200	1.00 [1.00;1.00]	0.691
PCT (L/L)	200	2.91 [0.03;250]	0.638
MPV (fl)	200	1.02 [0.77;1.34]	0.912
SD value of PDW (fl)	200	1.05 [0.48;2.27]	0.911
Platelet-large cell ratio (%)	200	1.00 [0.96;1.04]	0.946
PT(s)	200	1.40 [0.98;1.98]	0.062
PTA (%)	200	0.98 [0.96;1.00]	0.084
FIB (g/L)	200	1.48 [1.12;1.94]	0.005
APTT(s)	200	1.02 [0.96;1.08]	0.552
D-dime (mg/L)	200	1.41 [0.94;2.12]	0.093
ALB (g/L)	200	0.92 [0.86;0.98]	0.015
GLO (g/L)	200	1.00 [0.98;1.02]	0.705
ALT (U/L)	200	1.00 [0.99;1.01]	0.962
AST (U/L)	200	1.01 [0.99;1.02]	0.485
RBG (mmol/L)	200	1.21 [1.05;1.40]	0.010
BUN (mmol/L)	200	1.02 [0.96;1.08]	0.583
Scr (umol/L)	200	0.99 [0.98;1.00]	0.115
BUN/Scr (%)	200	1.04 [1.00;1.08]	0.036
UA (umol/L)	200	1.00 [0.99;1.00]	0.013
K (mmol/L)	200	1.32 [0.70;2.51]	0.394
NA (mmol/L)	200	0.90 [0.81;1.00]	0.049
CL (mmol/L)	200	0.99 [0.95;1.03]	0.665
TC (mmol/L)	200	1.05 [0.76;1.45]	0.772
TG (mmol/L)	200	0.84 [0.60;1.17]	0.303
HDL-C (mmol/L)	200	0.72 [0.17;3.08]	0.656
LDL-C (mmol/L)	200	1.11 [0.72;1.71]	0.644
APO-AI (g/L)	200	1.09 [0.81;1.45]	0.575
APO-B (g/L)	200	1.07 [0.84;1.37]	0.582
Lpa (mmol/L)	200	1.00 [1.00;1.00]	0.208
CRP (mg/L)	200	1.03 [1.00;1.05]	0.059
HCY (umol/L)	200	0.99 [0.93;1.06]	0.837
CK (u/L)	200	1.00 [1.00;1.00]	0.697
CK-MB (u/L)	200	1.00 [0.96;1.04]	0.951
LDH (u/L)	200	1.00 [1.00;1.01]	0.163

WBC, white blood cell; N, neutrophil; RBC, red blood cell; Hb, Hemoglobin; Hct, Hematocrit; MCV, mean corpuscular volume; MCH, mean corpuscular hemoglobin; MCHC, mean corpuscular hemoglobin concentration; SD, value of red blood cell volume distribution width; PLT, platelet; PCT, platelet accumulation; MPV, mean platelet volume; PT, prothrombin time; PTA, prothrombin activity; FIB, fibrin; APTT, activated partial thromboplastin time; ALB, serum albumin; GLO, serum globulin; ALT/GPT, glutamic pyruvic transaminase (GPT); AST/GOT, Glutamic oxaloacetic transaminase; RBG, random blood glucose; BUN, blood urea nitrogen; Scr, creatinine; UA, uric acid; K, calcium; NA, natrium; CL, chlorine; TC, total cholesterol; TG, triglyceride; HDL, high-density lipoprotein; LDL, low-density lipoprotein; APO-AI, apolipoprotein AI; APO-B apolipoprotein B; Lpa, lipoprotein B; CRP, C-reactive protein; HCY, homocysteine; CK, creatine kinase; CK-MB, creatine kinase isoenzyme; LDH, lactic dehydrogenase.

### 3.3 The nomogram to predict the outcomes of patients after stroke in week 3 follow-up

**Figure 1:**
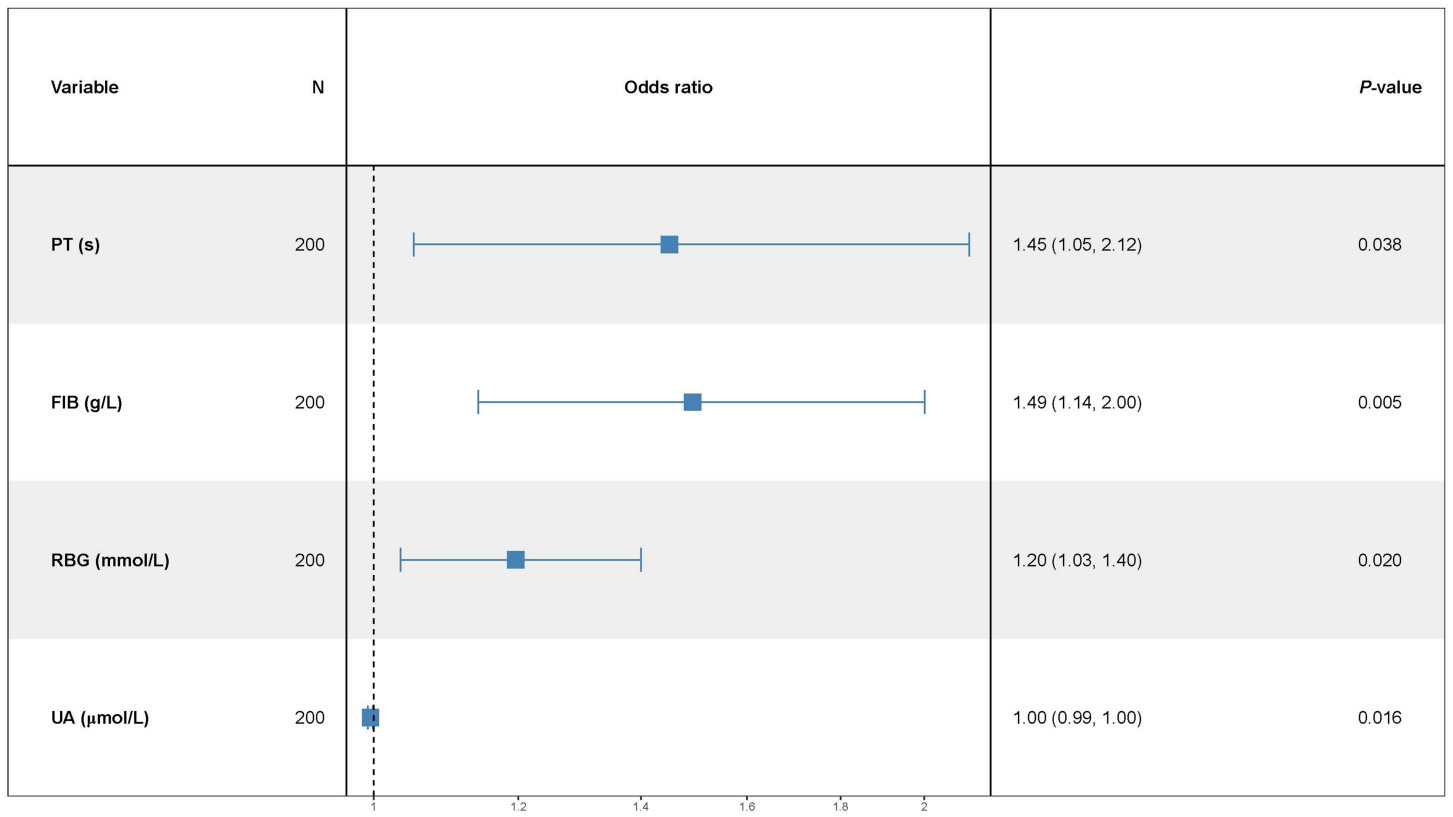
Independent predictors of functional outcome. ORs (relative risk) and 95% CIs are shown.

**Figure 2:**
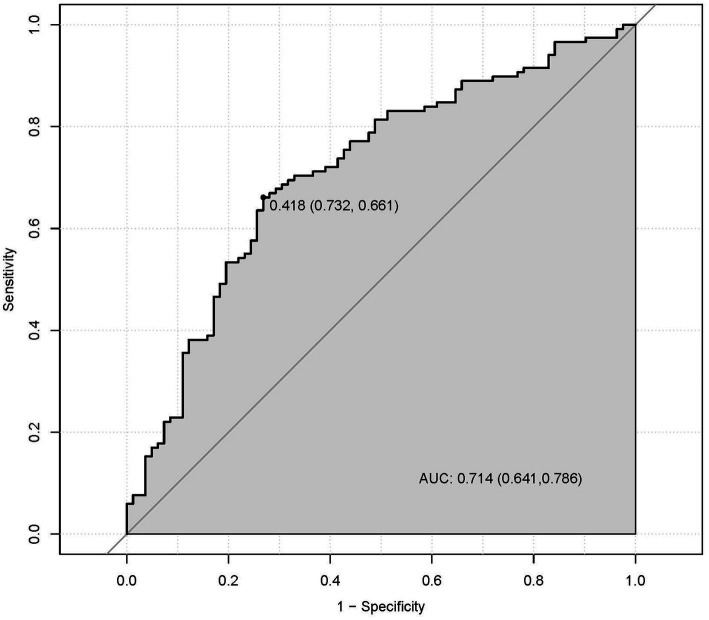
The discriminatory capacity of related factors, including PT, FIB, RBG, and UA for distinguishing between patients with the good functional outcomes and bad outcomes.

A multivariate predictive model was established, including PT(s), FIB, RBG, and UA (Figure 1). The statistically significant variables were used to construct the predictive nomogram model (Figure 2). The ROC curve was used to assess the model’s predictive accuracy, and a higher AUC value of 0.714 (95%CI: 0.641–0.786) was found to distinguish between good and poor outcomes after stroke (Figure 2).

**Figure 3:**
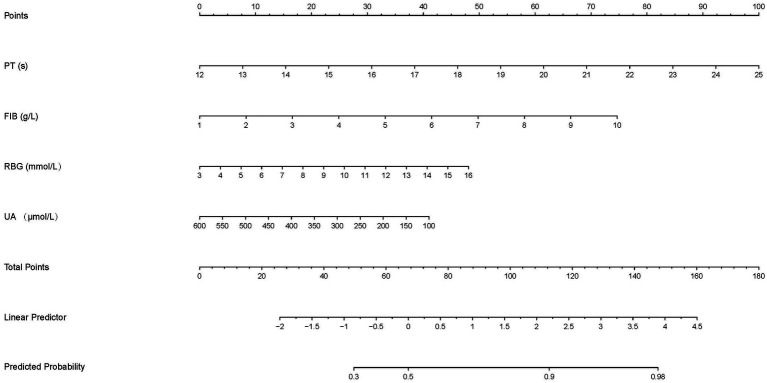
The nomogram to predict the probability of the functional outcomes in stroke patients. Each factor was given a point on the basis of the nomogram. The final total points were obtained by adding the individual score of each of the four related factors. The estimated probability of functional outcomes can easily be obtained from the nomogram based on the total points.

**Figure 4:**
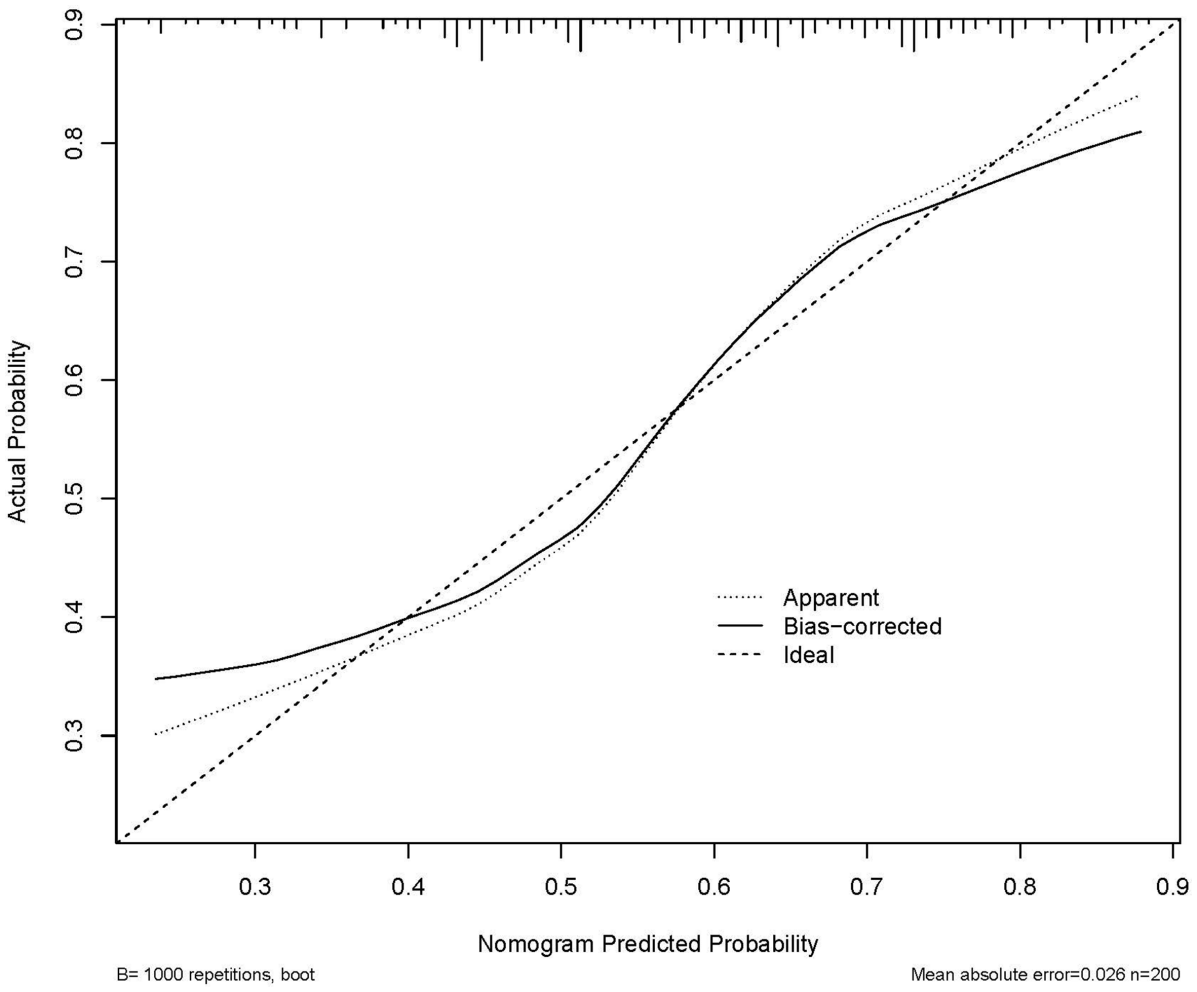
Calibration plot of the nomogram in 3-week functional outcomes in patients. The 45° line in the plot indicates a perfect calibration that the predictive capability of the model perfectly matches the actual risk of the functional outcomes. The dotted line represents the performance of the nomogram, whilst the solid line corrects for any bias in the nomogram.

The nomogram predicted probability of PT(s), FIB, RBG, and UA in the outcomes after stroke was obtained using the logistic regression analysis (Figure 3). Calibration curves using the bootstrap method (1,000 times) were plotted (Figure 4), showing good agreement between the predicted models and the actual observations.

**Figure 5:**
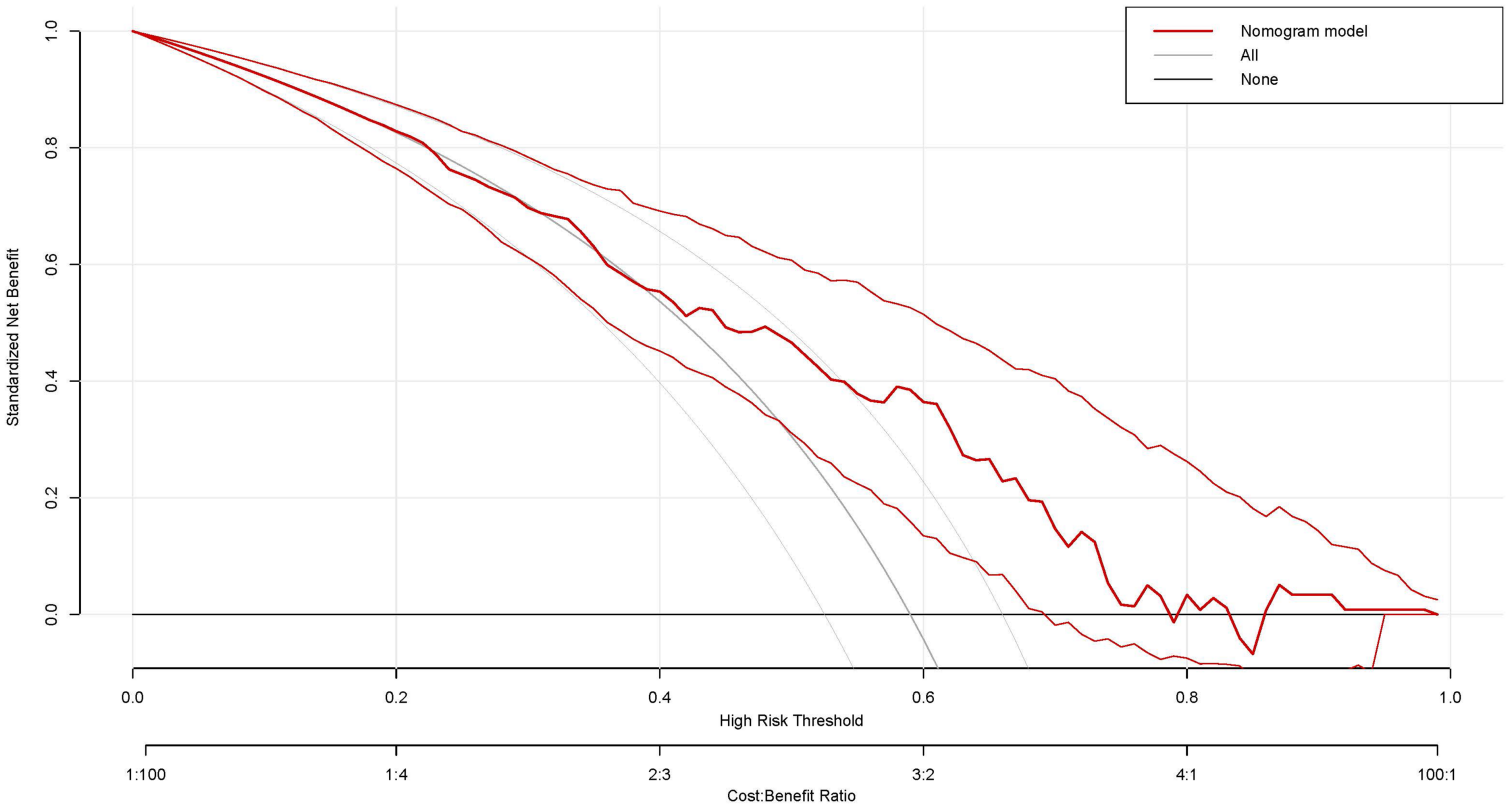
Decision curve analysis of the nomogram in this study. *x*-axis, the threshold probability; y-axis, the net benefit. The grey line indicates that all stroke patients will develop bad functional outcomes. The black line indicates that no stroke patients will develop bad functional outcomes. The red line is the nomogram to predict the functional outcomes in patients with stroke.

Subsequently, a comprehensive evaluation of PT(s), FIB, RBG, and UA was conducted using the decision curve analysis (DCA). Notably, this nomogram prediction model showed high net clinical benefit, which is shown in Figure 5. In summary, our nomogram prediction model demonstrated optimal performance in predicting outcomes in stroke patients after 3 weeks, not only in terms of statistical superiority, but also in terms of its utility and decision-support capabilities in clinical practice.

## 4 Discussion

Stroke may lead to serious and long-term disability. Early and accurate prediction of outcomes after a stroke, especially within the critical first few weeks, is critical for choosing appropriate therapy and rehabilitation strategies and managing patient care effectively. In this study, we found that stroke outcomes assessed using the BI scales were related to age, literacy levels, history of stroke, and disease courses.

In previous studies, age has tended to be related to higher risks of stroke and poorer outcomes due to age-associated alterations in the brain and body (Nakayama et al., 1994; Bagg et al., 2002; Roy-O’Reilly and McCullough, 2018; Ouyang et al., 2024). Literacy levels can affect a patient’s ability to understand and follow rehabilitation instructions, which is crucial for recovery (Meherali et al., 2020). A history of previous stroke may also indicate a higher likelihood of recurrent stroke and potentially more severe outcomes (Wang et al., 2011; Sharma et al., 2019). The course of the disease, including the severity of the stroke and the presence of complications, will also play a significant role in determining a patient’s functional abilities and quality of life after a stroke (Pien et al., 2023). All these studies underscore the importance of considering these factors when assessing and predicting stroke outcomes.

In addition to the demographic and clinical data, we found that a number of biomedical indicators, such as WBC, PT, FIB, ALB, RBG, BUN/Scr(%), and UA were associated with the stroke outcomes at 3 weeks after stroke. WBC is a component of blood tests that can indicate the presence of infection or inflammation, which may complicate stroke recovery. In one study, PT, along with the systemic Inflammatory Response Index (SIRI) and the Systemic Immune-Inflammation Index (SII), were reported to be a prognostic factor in stroke (Zhao et al., 2023). Higher PT, SIRI, and SII values were revealed to be independently related to poor outcomes at 3 months of acute ischemic stroke. Elevated levels of FIB, a protein that plays a role in blood clotting, may be associated with an increased risk of thrombosis, a common cause of stroke (Di Napoli et al., 2001; Rothwell et al., 2004; Turaj et al., 2006). ALB can help to maintain fluid balance in the body, and low concentrations may suggest malnutrition or liver dysfunction, which can affect stroke outcomes (Dziedzic et al., 2004; Ginsberg et al., 2006). A multicenter prospective cohort study has reported that the ratio of BUN/Scr, used to assess kidney function was associated with all-cause mortality (Inaguma et al., 2018). Considering that kidney disease can influence the body’s ability to clear waste products, the ratio of BUN/Scr, as a risk of stroke may be associated with the prognosis after stroke. UA levels were associated with metabolic disorders and oxidative stress (Waring, 2002; Cortese et al., 2020; Serdarevic et al., 2020), which may be involved in stroke pathophysiology.

These studies underscore the importance of considering biomedical indicators, along with demographic and clinical data, when assessing and predicting stroke outcomes. Interestingly, after adding all demographic data, clinical data, and biomedical indicators as independent variables, the nomogram provided a comprehensive predictive model. It predicted that PT, FIB, RBG, and UA were correlated with 3-week functional outcomes after stroke in patients with satisfactory discrimination and calibration ability. It is well-known that the first 8 weeks after a stroke are critical for recovery, as most recovery occurs during this period (Wade et al., 1985; Liu et al., 2022). Tailored rehabilitation requires a personalized approach and predictive biomarkers (Cacciotti et al., 2024). Notably, previous studies have reported many potential biomarkers related to poorer outcomes after stroke (De Marchis et al., 2013; Wang et al., 2014), including inflammation, atherogenesis, and stress response (Tang et al., 2017; Blek et al., 2022). Although the development of novel predictive models has made significant progress in the prediction of functional outcomes after stroke, there is still a need to discover additional markers, particularly to provide further evidence for future work in deep learning and multimodal data and feature selection. Our study provides further evidence to use nomograms to predict potentially the prognosis of stroke patients, as assessed by the BI scale. Previous studies have developed nomogram prediction models to identify stroke patients who are likely to show improved functional outcomes following rehabilitation (Chen and Zhang, 2024; Yang et al., 2024), indicating that nomograms can be used to predict the outcomes of patients. A study identified independent risk factors for poor prognosis in patients with cerebral infarction, such as gender, smoking, drinking, lack of exercise, and post-discharge use of biguanide hypoglycemic drugs by constructing a personalized prediction model based on a nomogram, which could help clinical decision-making (Chen et al., 2024). In addition, a nomogram was developed to predict short-term mortality using a variety of independent risk factors, which was found to be highly accurate in predicting the prognosis of patients with stroke (Jin et al., 2023). All these studies suggest that nomograms can be valuable tools in clinical practice. Our study provides a novel predictive model combining the following biomarkers: PT, FIB, RBG, and UA, which can help to more accurately predict functional outcomes at 3 weeks post-stroke, especially in assessing the individual’s ADL ability. The predicting model of these four predicated biomarkers may help healthcare providers make decisions about prevention strategies and treatment plans for patients.

Several limitations should be noted in our study. First, retrospective studies may be subject to selection bias. In retrospective studies, data collection may rely on the completeness and accuracy of medical records, which may lead to information bias. Retrospective studies are usually unable to establish causal relationships because they cannot control experimental conditions as effectively as randomized controlled trials. The results of retrospective studies were from specific populations or medical centers and may not be generalizable or easily extrapolated to other populations or environments. Second, nomograms derived from retrospective studies need to be validated in independent prospective studies to confirm the predictive performance. Further study in a cohort of stroke patients is warranted to verify our findings. Third, it has been suggested that biomarkers fluctuate during hospitalization. Therefore, it is essential that future research on stroke includes a dynamic assessment of these biomarkers, which may track their variations over time and potentially understand the potential role in the progression and prognosis of stroke. Dynamic monitoring may provide valuable insights into the pathophysiology of stroke and aid in the development of more effective treatment strategies.

## 5 Conclusion

This study developed an innovative nomogram that incorporated four blood biomarkers, including PT, FIB, RBG, and UA, for predicting functional outcomes at 3 weeks post-stroke. The nomogram prediction model could provide valuable insights to neurologists by assisting in the prognostic assessment of stroke patients. Our findings may lead to more personalized and effective clinical interventions in the management of stroke patients. However, further studies are needed to enhance the nomogram’s ability to accurately discriminate and calibrate predictions, thereby improving its predictive accuracy for patient outcomes.

## Data Availability

The raw data supporting the conclusions of this article will be made available by the authors, without undue reservation.
